# Low titers of SARS-CoV-2 neutralizing antibodies after first vaccination dose in cancer patients receiving checkpoint inhibitors

**DOI:** 10.1186/s13045-021-01099-x

**Published:** 2021-05-31

**Authors:** Evangelos Terpos, Flora Zagouri, Michalis Liontos, Aimilia D. Sklirou, Konstantinos Koutsoukos, Christos Markellos, Alexandros Briasoulis, Eleni-Dimitra Papanagnou, Ioannis P. Trougakos, Meletios-Athanasios Dimopoulos

**Affiliations:** 1 Department of Clinical Therapeutics, School of Medicine, Alexandra General Hospital, National and Kapodistrian University of Athens, 80 Vas. Sofias Avenue, 11528 Athens, Greece; 2 Department of Cell Biology and Biophysics, Faculty of Biology, National and Kapodistrian University of Athens, Athens, Greece

**Keywords:** SARS-CoV-2, Vaccination, Cancer, Immune checkpoint inhibitors, BNT162b2, AZD1222

## Abstract

Vaccination for SARS-CoV-2 provides significant protection against the infection in the general population. However, only limited data exist for patients with cancer under systemic therapy. Based on this, our site has initiated a study evaluating safety and efficacy of SARS-CoV-2 vaccination in patients with solid and hematological malignancies under several systemic therapies. The initial results of the cohort of 59 patients receiving Immune Checkpoint Inhibitors are presented here. Despite no new safety issues have been noticed, the levels of SARS-CoV-2 neutralizing antibodies are significantly lower in comparison to matched healthy volunteers up to day 22 post the first dose. These results should be taken into consideration for the patients under treatment.

**Supplementary Information:**

The online version contains supplementary material available at 10.1186/s13045-021-01099-x.

To the Editor,

Patients with cancer are considered vulnerable to SARS-CoV-2 infection [1] and have been prioritized in the vaccination process in several countries including Greece. In addition, international oncological societies favored COVID-19 vaccination for cancer patients on the basis of risk and benefits evaluation of all available data. However, patients with cancer were excluded from SARS-CoV-2 vaccines registrational trials [2, 3] and we lack data regarding the safety and efficacy of vaccination in this population. Under this perspective, we undertook a large prospective study (NCT04743388) enrolling patients with solid cancers, hematologic malignancies as well as healthy volunteers for the kinetics of anti-SARS-CoV-2 antibodies after COVID-19 vaccination [4]. Herein, we report the development of neutralizing antibodies (NAbs) against SARS-CoV-2 in patients with solid tumors receiving immune checkpoint inhibitors (ICIs) after the first dose of the BNT162b2 and AZD1222 vaccines. Major inclusion criteria for this cohort of the study included: (1) age above 18 years; (2) presence of solid organ malignancies treated with immunotherapy irrespective of the treatment phase; and (3) eligibility for vaccination.

The serum of both patients and controls was collected on day 1 prior to vaccination and on day 22. NAbs against SARS-CoV-2 were measured using FDA approved methodology (ELISA, cPass™ SARS-CoV-2 NAbs Detection Kit; GenScript, Piscataway, NJ, USA) [5] on the abovementioned timepoints. Samples of the same patient or control were measured in the same ELISA plate. The study was approved by the respective Ethical Committees in accordance with the Declaration of Helsinki and the International Conference on Harmonization for Good Clinical Practice. All patients and controls provided written informed consent prior enrollment in the study. Baseline demographics, co-morbidities, and the NAb levels were compared between the 2 groups, Chi-square test for categorical variables and unpaired t-test or Wilcoxon signed-rank test (as appropriate) for continuous variables. To adjust for potential confounding effects of differences in covariates, we used case–control matching to match the two groups for age, gender, and type of vaccine with the calipmatch command in Stata. All data extraction and analyses were conducted using Stata 16.0 (StataCorp 2019, Stata Statistical Software: Release 16. College Station, TX: StataCorp LLC). Two-sided *p* value < 0.05 was used for statistical significance.

Study population included 59 patients (36 males/23 females; median age: 66 years, IQR 61–76 years) and 283 controls (median age: 64 years, IQR 59–82 years, *p* = 0.75 for age compared with patients), vaccinated during the same period. 44/59 patients (74.6%) and 232/283 controls (82%) were vaccinated with a mRNA vaccine (BNT162b2 or mRNA-1273), while the remaining received the AZD1222 vaccine (*p* = 0.19). The characteristics of the patients are depicted in Table 1. Among patients, 16 had lung cancer, 15 bladder cancer, 11 kidney carcinoma, and the remaining 17 other carcinomas. Most patients (49 patients, 83.0%) received anti-PD1 treatment, while 10 (17.0%) received anti-PD-L1 antibodies or immunotherapy combination. Comorbidities in the patient group included cardiovascular disease in 42.4%, diabetes in 20.3%, pulmonary disease in 10.2%.

**Table 1:** Characteristics of the patients

Characteristic	Total population
	Median (IQR)
Age	66 (61–76)
BMI	26.1 (23.6–28.3)
	*N* (%)
Sex	
Male	36 (61.0%)
Female	23 (39.0%)
Type of cancer	
Lung cancer	16 (27.1%)
Bladder cancer	15 (25.4%)
Kidney cancer	11 (18.6%)
Uterine cancer	5 (8.5%)
Pancreatic cancer	3 (5.1%)
Other	8 (13.6%)
Missing	1 (1.7%)
Type of therapy	
Anti-PD1	49 (83.0%)
Anti-PD-L1	8 (13.6%)
I/O combo	2 (3.4%)
Vaccine	
BNT162b2	41 (69.5%)
AZD1222	15 (25.4%)
mRNA-1273	3 (5.1%)
Vaccine-related adverse events	
None	37 (62.7%)
Fever	1 (1.7%)
Pain at injection site	11 (18.6%)
Fatigue	3 (5.1%)
Other	1 (1.7%)
Comorbidities	
Yes	39 (66.1%)
None	11 (18.6%)
Missing	9 (15.3%)

*IQR* interquartile range, *BMI* body mass index, *I/O* Immunotherapy

On D1, two patients (3.4%) and 26 (9.2%) controls had NAb titers of ≥ 30% (positivity cut-off); there was no difference regarding the NAb titers between patients and controls on D1 (*p* = 0.35). None of them had a prior history of known COVID-19. After the first vaccine dose, on D22, patients had lower NAb titers compared to controls: the median NAb inhibition titer was 22% (IQR 13.4–30.2%) for patients versus 38% (IQR 23–54%) for controls; *p* < 0.001 (Fig. 1). More, specifically, 15 (25%) patients versus 186 (65.7%) controls developed NAb titers ≥ 30% on D22 ( *p* <  0.001). The respective number of patients and controls who developed NAb titers ≥ 50% (clinically relevant viral inhibition [6]) was 6 (10.7%) and 93 (32.9%), respectively (*p* = 0.01). Of note, none of the patients enrolled had neutropenia or lymphopenia at first vaccination dose (Additional file 1: Table S1).

**Fig. 1:**
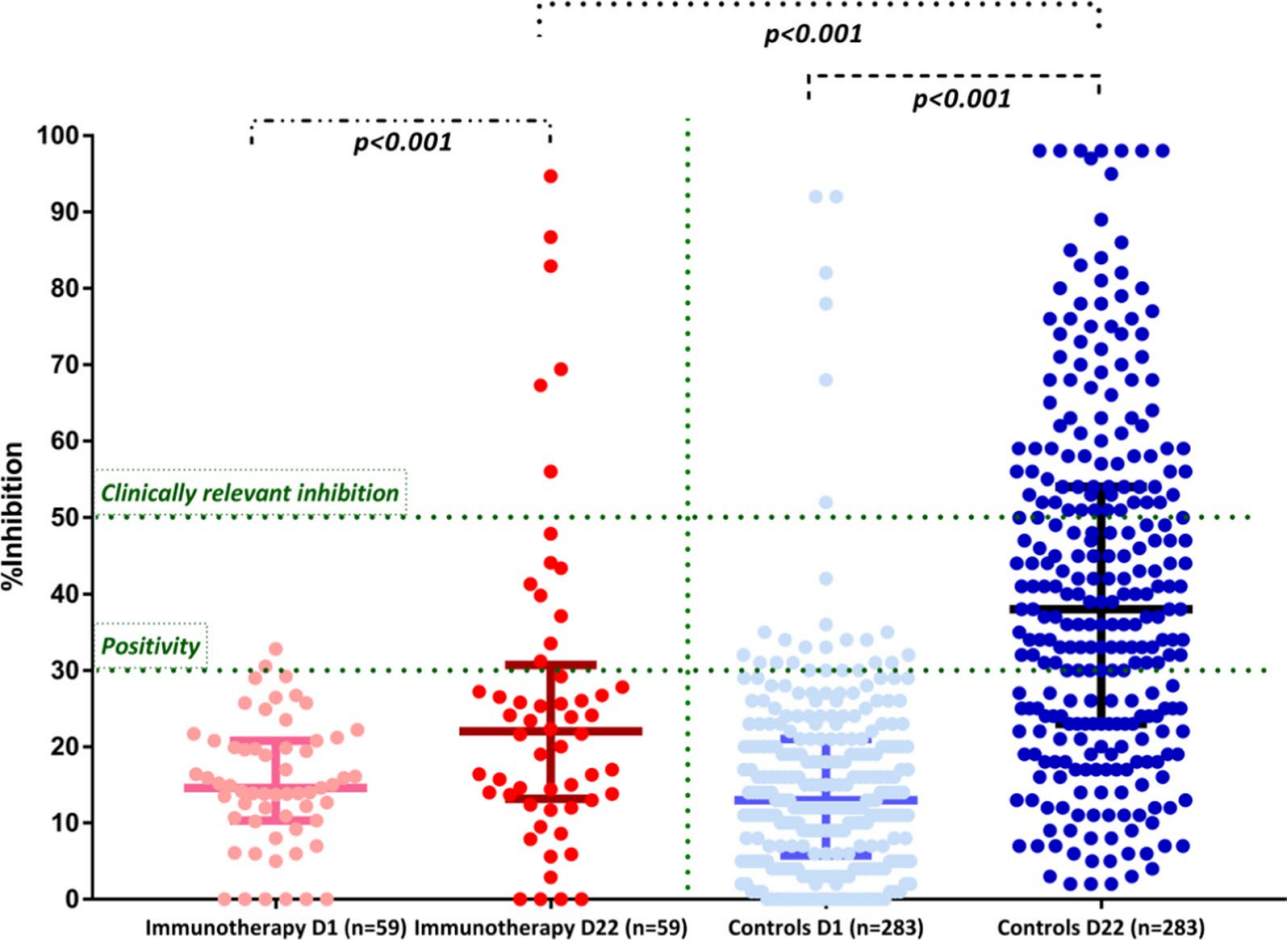
Kinetics of neutralizing antibodies in patients receiving immunotherapy and matched controls after vaccination with the first dose of the BNT162b2, mRNA-1273 and AZD1222 vaccine. On D22, patients had lower production of NAb inhibition titers compared to controls of similar age and gender (see text). Only 6 patients (10.7%) had NAb titers of equal or more than 50%

Recently, Waissengrin et al. [7] reported the safety results of BNT162b2 vaccine in patients with cancer treated with ICIs. We confirm these data in our study population; amongst the 59 patients of our department who received vaccination while on treatment with ICIs, no unexpected adverse events were noted. During the post vaccination follow-up period (median 44 days, IQR 36–67 days) immunotherapy related adverse events were recorded in one patent (1.7%). For the first time, we also report that patients on treatment with ICIs receiving the first dose of the BNT162b2 and AZD1222 vaccines develop low titers of NAb against SARS-CoV-2 by day 22. These results could be attributed to the immunosuppressive effect of cancer and/or treatment given and inform regarding the optimal management of these patients at least until vaccination completion. Further follow-up of the current study will provide significant data for the efficacy of vaccination in cancer patients.

## Supplementary Information

**Additional file 1. Table S1:** Detailed characteristics of patients

## Data Availability

All data generated or analysed during this study are included in this published article and its supplementary information file.

## Abbreviations

ICIs: Immune checkpoint inhibitors
NAbs: Neutralizing antibodies
IQR: Interquartile range
