# Supplementary material for: Low titers of SARS-CoV-2 neutralizing antibodies after first vaccination dose in cancer patients receiving checkpoint inhibitors
Source: J Hematol Oncol. 2021 May 31;14:86. doi: 10.1186/s13045-021-01099-x (PMC8165511; doi:10.1186/s13045-021-01099-x)
Supplement: Supplementary file 1 — Additional file 1. Table S1: Detailed characteristics of patients [file 13045_2021_1099_MOESM1_ESM.docx]

| **Additional file 1: Table S1. Detailes characteristics of patients** | | | | | | | | |  | |  | |  | |  | |  | |  | |
| --- | --- | --- | --- | --- | --- | --- | --- | --- | --- | --- | --- | --- | --- | --- | --- | --- | --- | --- | --- | --- |
|  |  |  |  |  |  |  |  |  | |  | |  | |  | |  | |  | |  |
| **#** | **SEX** | **AGE** | **BMI** | **TYPE OF CANCER** | **TYPE OF THERAPY** | **MONTHS ON TREATMENT** | **COMORBIDITIES** | **VACCINE** | | **ADVERSE EVENTS** | | **NEUTROPHILES** | | **LYMPHOCYTES** | | **irAEs** | | **CORTICOSTEROIDS USE** | |  |
| 1 | F | 89 | 22,7 | Bladder cancer | Anti-PD1 | 2 | Hypertension, Hypothyroidism | BNT162b2 | | None | | 7250 | | 1940 | | None | | No | |  |
| 2 | Μ | 88 | 19,1 | Bladder cancer | Anti-PD-L1 | 45 | Hypertension, Stroke | BNT162b2 | | Pain at the site of injection | | 4665 | | 3290 | | None | | No | |  |
| 3 | Μ | 87 | 27,7 | Lung cancer | Anti-PD-L1 | 3 | NA | BNT162b2 | | None | | 5400 | | 1760 | | None | | No | |  |
| 4 | F | 81 | 21,3 | Pancreatic cancer | Anti-PD1 | 6 | Hypertension, Atrial Fibrillation | BNT162b2 | | Pain at the site of injection | | 3840 | | 2000 | | None | | No | |  |
| 5 | Μ | 61 | 30,4 | Ampullary cancer | Anti-PD1 | 32 | Hypertension, Coronary artery disease, Diabetes Mellitus | AZD1222 | | None | | 4500 | | 3400 | | None | | No | |  |
| 6 | Μ | 64 | 31,0 | Bladder cancer | Anti-PD1 | 13 | Hypertension | AZD1222 | | Pain at the site of injection | | 6000 | | 2900 | | None | | No | |  |
| 7 | Μ | 75 | 24,3 | Bladder cancer | Anti-PD1 | 8 | Hypertension, Diabetes Mellitus | BNT162b2 | | None | | 5040 | | 3200 | | None | | No | |  |
| 8 | Μ | 75 | 27,4 | Kidney cancer | Anti-PD1 | 10 | None | BNT162b2 | | None | | NA | | NA | | None | | No | |  |
| 9 | Μ | 62 | 26,5 | Kidney cancer | Anti-PD1 | 1 | Coronary artery disease, Diabetes Mellitus | AZD1222 | | Pain at the site of injection | | 6500 | | 3100 | | Hypophsyitis | | No | |  |
| 10 | Μ | 82 | 23,7 | Bladder cancer | Anti-PD1 | 6 | None | BNT162b2 | | None | | 7950 | | 2800 | | None | | No | |  |
| 11 | Μ | 62 | 30,9 | Bladder cancer | Anti-PD-L1 | 66 | None | AZD1222 | | None | | 4680 | | 1200 | | None | | No | |  |
| 12 | Μ | 71 | NA | Lung cancer | Anti-PD1 | NA | NA | BNT162b2 | | Pain at the site of injection | | NA | | NA | | None | | No | |  |
| 13 | F | 62 | 37,0 | Uterine cancer | Anti-PD1 | 15 | Asthma | AZD1222 | | None | | 4100 | | 3100 | | None | | No | |  |
| 14 | Μ | 64 | 31,2 | Kidney cancer | Anti-PD1/anti-CTLA4 | 4 | Parkinson's Disease | AZD1222 | | None | | 3470 | | 2450 | | None | | No | |  |
| 15 | Μ | 64 | 16,5 | Lung cancer | Anti-PD1 | 13 | None | AZD1222 | | None | | 1980 | | 1800 | | None | | No | |  |
| 16 | Μ | 81 | 27,5 | Bladder cancer | Anti-PD1 | 11 | Hypertension, Diabetes Mellitus | BNT162b2 | | None | | 5450 | | 1450 | | None | | No | |  |
| 17 | F | 60 | 24,0 | Lung cancer | Anti-PD1 | 2 | Dyslipidemia | AZD1222 | | None | | 1400 | | 2740 | | None | | No | |  |
| 18 | Μ | 86 | 27,1 | Merckel cell carcinoma | Anti-PD-L1 | 1 | COPD | BNT162b2 | | None | | NA | | NA | | None | | No | |  |
| 19 | F | 84 | 28,0 | Lung cancer | Anti-PD1 | NA | Hypertension, Atrial Fibrillation | BNT162b2 | | None | | 2800 | | 2000 | | None | | Yes | |  |
| 20 | F | 76 | NA | Lung cancer | Anti-PD1 | 67 | None | BNT162b2 | | None | | 5200 | | 1500 | | None | | No | |  |
| 21 | Μ | 63 | 28,0 | Kidney cancer | Anti-PD1 | 4 | Hypertension | AZD1222 | | Pain at the site of injection | | 3290 | | 1200 | | None | | No | |  |
| 22 | F | 78 | NA | Lung cancer | Anti-PD1 | 16 | Hypertension, Diabetes Mellitus, Dyslipidemia, Hypothyroidism | BNT162b2 | | None | | 3900 | | 1200 | | None | | No | |  |
| 23 | Μ | 64 | 25,8 | Kidney cancer | Anti-PD1 | 26 | Hypothyroidism | AZD1222 | | None | | NA | | NA | | None | | No | |  |
| 24 | Μ | 76 | 24,7 | Bladder cancer | Anti-PD1 | 5 | Diabetes Mellitus, Dyslipidemia | BNT162b2 | | None | | 9820 | | 2700 | | None | | No | |  |
| 25 | Μ | 62 | 25,6 | Lung cancer | Anti-PD1 | NA | Hypertension, Dyslipidemia | AZD1222 | | None | | 7910 | | 2196 | | None | | No | |  |
| 26 | M | 64 | NA | Kidney cancer | Anti-PD1 | 4 | NA | AZD1222 | | Pain at the site of injection | | NA | | NA | | None | | No | |  |
| 27 | Μ | 77 | 26,0 | Bladder cancer | Anti-PD1 | 1 | COPD | BNT162b2 | | None | | 3970 | | 3100 | | None | | No | |  |
| 28 | Μ | 79 | 22,8 | Bladder cancer | Anti-PD1 | 67 | None | BNT162b2 | | None | | 4700 | | 1800 | | None | | No | |  |
| 29 | Μ | 62 | 27,9 | Bladder cancer | Anti-PD1 | 4 | Hyperthyroidism, Hepatitis, Stroke | AZD1222 | | None | | 3770 | | 2170 | | None | | No | |  |
| 30 | Μ | 70 | 23,5 | Bladder cancer | Anti-PD1/anti-CTLA4 | 19 | Coronary artery disease, Diabetes Mellitus | BNT162b2 | | None | | 5310 | | 2510 | | None | | No | |  |
| 31 | Μ | 63 | 29,0 | Kidney cancer | Anti-PD1 | 9 | Hypertension, Deep Vein Thrombosis, Hyperthyroidism | AZD1222 | | None | | 4290 | | NA | | None | | No | |  |
| 32 | M | 65 | 27,2 | Gastric cancer | Anti-PD1 | 3 | Hypertension, Dyslipidemia, COPD, Diabetes | BNT162b2 | | None | | 6510 | | 900 | | None | | No | |  |
| 33 | F | 62 | 22,9 | Lung cancer | Anti-PD1 | 6 | Hypothyroidism | AZD1222 | | NA | | 2300 | | 4300 | | None | | No | |  |
| 34 | F | 59 | 44,8 | Uterine cancer | Anti-PD1 | 7 | Osteoarthritis | BNT162b2 | | Fatigue | | NA | | NA | | None | | No | |  |
| 35 | F | 68 | 20,7 | Lung cancer | Anti-PD1 | 36 | None | BNT162b2 | | NA | | 3810 | | 2100 | | None | | No | |  |
| 36 | M | 70 | 37,5 | Bladder cancer | Anti-PD1 | 1 | Benign prostate hyperplasia, peptic ulcer, hyperuricemia, diabetes mellitus | mRNA-1273 | | Arthralgia, Fatigue | | 5200 | | 1840 | | None | | No | |  |
| 37 | Μ | 59 | 29,1 | Pancreatic cancer | Anti-PD1 | 6 | Hypertension, Diabetes Mellitus | BNT162b2 | | None | | 5500 | | 4900 | | None | | Yes | |  |
| 38 | F | 69 | 25,3 | Uterine cancer | Anti-PD1 | 2 | Hypertension | BNT162b2 | | Pain at the site of injection | | 6800 | | 2200 | | None | | No | |  |
| 39 | F | 65 | NA | Cancer of Unkown Origing | Anti-PD1 | 3 | NA | BNT162b2 | | Pain at the site of injection | | NA | | NA | | None | | No | |  |
| 40 | F | 75 | 21,1 | Uterine cancer | Anti-PD1 | 6 | Hypertension, dyslipidemia, Arrhythmias, Diabetes | BNT162b2 | | None | | 4150 | | 1600 | | None | | No | |  |
| 41 | F | 85 | 23,4 | Kidney cancer | Anti-PD1 | 16 | Pulmonary embolism, hypertension | mRNA-1273 | | NA | | 3980 | | 750 | | None | | No | |  |
| 42 | Μ | 72 | 23,6 | Pancreatic cancer | Anti-PD1 | 18 | Hypertension, glaucoma | BNT162b2 | | None | | 2100 | | 3100 | | None | | No | |  |
| 43 | F | 73 | 44,9 | Bladder cancer | Anti-PD1 | 9 | Dyslipidemia | BNT162b2 | | None | | 4950 | | 3650 | | None | | No | |  |
| 44 | M | 53 | 30,6 | Kidney cancer | Anti-PD1 | 7 | None | BNT162b2 | | Pain at the site of injection, fever | | 5310 | | 2480 | | None | | No | |  |
| 45 | F | 49 | 38,1 | Uterine cancer | Anti-PD1 | 2 | NA | BNT162b2 | | None | | NA | | NA | | None | | No | |  |
| 46 | F | 51 | 20,6 | Bladder cancer | Anti-PD1 | 8 | Gestational Trophoblastic Disease | BNT162b2 | | None | | NA | | NA | | None | | No | |  |
| 47 | F | NA | NA | Lung cancer | Anti-PD-L1 | NA | NA | BNT162b2 | | NA | | NA | | NA | | None | | No | |  |
| 48 | Μ | 53 | 25,5 | Lung cancer | Anti-PD1 | 5 | Stroke | BNT162b2 | | None | | 3700 | | 2800 | | None | | No | |  |
| 49 | M | 71 | 25,2 | Lung cancer | Anti-PD1 | 36 | Hypertension, Coronary artery disease, Aortic aneurysm | BNT162b2 | | None | | 5750 | | 3100 | | None | | No | |  |
| 50 | F | 59 | 24,5 | Lung cancer | Anti-PD-L1 | 3 | Hypertension, asthma, diabetes mellitus, hypothyroidism | BNT162b2 | | None | | 1550 | | 450 | | None | | No | |  |
| 51 | F | 41 | NA | Breast cancer | Anti-PD-L1 | 2 | NA | mRNA-1273 | | None | | NA | | NA | | None | | No | |  |
| 52 | Μ | 66 | 42,2 | Kidney cancer | Anti-PD1 | 11 | Pulmonary embolism, hypothyroidism, seizures | BNT162b2 | | None | | 2780 | | 1650 | | None | | No | |  |
| 53 | Μ | 60 | 26,1 | Lung cancer | Anti-PD1 | 1 | Arrhythmias,hyperuricemia,nephrolithiasis | BNT162b2 | | NA | | 3100 | | 3690 | | None | | No | |  |
| 54 | F | 58 | 23,8 | Breast cancer | Anti-PD-L1 | NA | None | BNT162b2 | | Facial rash | | 2300 | | 1300 | | None | | No | |  |
| 55 | M | 73 | 27,3 | Kidney cancer | Anti-PD1 | 15 | NA | BNT162b2 | | None | | 4330 | | 2100 | | None | | No | |  |
| 56 | F | 47 | NA | Cervical | Anti-PD1 | NA | NA | BNT162b2 | | NA | | NA | | NA | | None | | No | |  |
| 57 | M | 43 | 19,9 | Lung cancer | Anti-PD1 | 4 | None | BNT162b2 | | None | | 3150 | | 1400 | | None | | No | |  |
| 58 | F | 55 | 27,4 | Ovarian cancer | Anti-PD1 | 3 | None | BNT162b2 | | Fatigue | | 3550 | | 910 | | None | | No | |  |
| 59 | M | 68 | 19,6 | Colon cancer | Anti-PD1 | 1 | Hypertension | BNT162b2 | | Pain at the site of injection | | 2300 | | 1830 | | None | | No | |  |
